# Reversal of murine alcoholic steatohepatitis by pepducin-based functional blockade of interleukin-8 receptors

**DOI:** 10.1136/gutjnl-2015-310344

**Published:** 2016-02-08

**Authors:** Verena Wieser, Timon E Adolph, Barbara Enrich, Athan Kuliopulos, Arthur Kaser, Herbert Tilg, Nicole C Kaneider

**Affiliations:** 1 Department of Internal Medicine I, Gastroenterology, Endocrinology & Metabolism, Medical University Innsbruck, Innsbruck, Austria; 2 Christian Doppler Research Laboratory for Gut Inflammation, Medical University Innsbruck, Innsbruck, Austria; 3 Division of Gastroenterology and Hepatology, Department of Medicine, Addenbrooke's Hospital, University of Cambridge, Cambridge, UK; 4 Center for Hemostasis and Thrombosis Research, Molecular Oncology Research Institute, Tufts Medical Center, Tufts University School of Medicine, Massachusetts, USA

**Keywords:** ALCOHOLIC LIVER DISEASE, IMMUNE-MEDIATED LIVER DAMAGE, INTERLEUKIN 8, LEUKOCYTES

## Abstract

**Objective:**

Alcoholic steatohepatitis is a life-threatening condition with short-term mortality up to 40%. It features hepatic neutrophil infiltration and blood neutrophilia, and may evolve from ethanol-induced breakdown of the enteric barrier and consequent bacteraemia. Signalling through CXCR1/2 G-protein-coupled-receptors (GPCRs), the interleukin (IL)-8 receptors, is critical for the recruitment and activation of neutrophils. We have developed short lipopeptides (pepducins), which inhibit post-ligand GPCR activation precisely targeting individual GPCRs.

**Design:**

Experimental alcoholic liver disease was induced by administering alcohol and a Lieber–DeCarli high-fat diet. CXCR1/2 GPCRs were blocked via pepducins either from onset of the experiment or after disease was fully established. Hepatic inflammatory infiltration, hepatocyte lipid accumulation and overall survival were assessed as primary outcome parameters. Neutrophil activation was assessed by myeloperoxidase activity and liver cell damage by aspartate aminotransferase and alanine aminotransferase plasma levels. Chemotaxis assays were performed to identify chemoattractant signals derived from alcohol-exposed hepatocytes.

**Results:**

Here, we show that experimental alcoholic liver disease is driven by CXCR1/2-dependent activation of neutrophils. CXCR1/2-specific pepducins not only protected mice from liver inflammation, weight loss and mortality associated with experimental alcoholic liver disease, but therapeutic administration cured disease and prevented further mortality in fully established disease. Hepatic neutrophil infiltration and triglyceride accumulation was abrogated by CXCR1/2 blockade. Moreover, CXCL-1 plasma levels were decreased with the pepducin therapy as was the transcription of hepatic IL-1β mRNA.

**Conclusions:**

We propose that high circulating IL-8 in human alcoholic hepatitis may cause pathogenic overzealous neutrophil activation, and therapeutic blockade via pepducins merits clinical study.

**Significance of this study**

**What is already known on this subject?**

- Neutrophilia in alcoholic hepatitis (AH) is associated with worse outcome.
- Expression of chemokines is massively increased in human AH.
- No therapies improving long-time survival exist.

**What are the new findings?**

- Blocking CXCR1/2 receptors increases survival in a murine model of alcoholic liver injury.
- CXCR1/2 pepducins revert steatosis and liver inflammation.
- Alcohol-induced liver injury is neutrophil mediated.
- CXCR1/2 pepducins can be used therapeutically.

**How might it impact on clinical practice in the foreseeable future?**

- The treatment of patients with acute alcoholic liver disease with CXCR1/2 blocking pepducins deserves evaluation in clinical trials.

## Objective

Morbidity and mortality due to excessive alcohol consumption is a major health problem worldwide affecting millions of people.^1^ The risk of alcohol-induced liver disease (ALD) increases proportionately with consumption, causing a spectrum of liver diseases ranging from steatosis to terminal liver disease and cirrhosis.^2^ At 6-month mortality rates up to 50%, the acutely deadliest manifestation of ALD is severe alcoholic hepatitis (AH).^2^ AH is estimated to affect 10–35% of heavy drinkers at some point in time.^3^ Severe AH exhibits a very specific presentation with hepatocyte steatosis, neutrophilic liver inflammation and necrosis, a characteristic peripheral blood neutrophilia, and manifests as liver failure including thrombocytopenia and coagulation disorders.^4^ Patients surviving severe AH have a high risk of developing fibrosis and cirrhosis, in turn increasing risk for hepatocellular carcinoma.^3^ No therapies exist that improve long-term survival of AH.^5^ ^6^ On this dire background, it has indeed been suggested that patients with severe AH should be routinely randomised into experimental trials.^7^

A common working model suggests that ethanol might induce inflammatory chemokines and cytokines via generation of reactive oxygen species and acetaldehydes,^8–10^ which may impact on endotoxin clearance in the liver. This, along with intestinal barrier dysfunction consequent to chronic alcohol exposure,^11^ leads to systemic endotoxaemia, whose extent indeed correlates with progression of liver disease,^12–14^ establishing a vicious circle of pro-inflammatory signalling.

Expression of chemokines that are involved in neutrophil recruitment and activation is massively increased in human AH.^15^ ^16^ Worse prognosis associates with blood neutrophilia,^2^ and elevated mRNA expression of interleukin (IL)-8 (CXCL8), CXCL5, Gro-γ (CXCL3) and CXCL6, all ligands of CXCR1/CXCR2 chemokine receptors.^15^ ^17^ ^18^ Multivariate analysis revealed hepatic IL-8 protein levels as an independent predictor of 90-day mortality in AH.^15^ Neutrophils release an array of bactericidal molecules that might cause tissue destruction.^19^ Despite these associative data, a causal role of neutrophils and/or CXCR1/2 signalling in severe AH has not yet been established.^3^ ^5^ ^14^

CXCR1 and CXCR2 are G protein-coupled receptors (GPCRs).^20^ GPCRs consist of seven transmembrane domains joined by three intracellular loops and a C-terminal domain (referred to as *i1-i4*) that are important for interaction with and activation of G proteins involved in signal transduction.^21^ ‘Pepducins’ are short peptides coupled to a lipid moiety that can be designed as agonists or antagonists with precise specificity in vitro and in vivo for a given GPCR.^22–26^ Pepducins integrate into the inner plasma membrane via a flip-flop mechanism and affect GPCR-dependent G-protein activation.^27–29^ We have reported pepducin ‘x1/2pal-i1’, specific for CXCR1 and CXCR2, which reverses established experimental systemic inflammatory response syndrome by inhibiting neutrophil activation.^25^ x1/2pal-i1 also abolishes tumour growth in a model of ovarian cancer^30^ and inhibits adenoma formation in *Apc^min/+^* mice.^31^

In an experimental model that phenocopies human AH, here we report that CXCR1/2 blockade cures established steatohepatitis, identifying a critical role of CXCR1/2 signalling and neutrophils in propagating this disease.

## Design

Pepducins directed against CXCR1/2 (x1/2pal-i1) and a nonsense-scrambled pepducin (scram-i1) were synthesised with carboxy-terminal amides by standard fMOC solid-phase methods at the Tufts University Core Facility (Tufts University, Boston, Massachusetts, USA) or purchased from Peptide 2.0 (Chantilly, Virginia, USA). Lieber–DeCarli diet (LDC) was purchased from Dyets (Bethlehem, Pennsylvania, USA). The IL-8 ELISA was from R&D systems, the SYBR-green PCR mix was purchased from Eurogentec (Southampton, UK). Naphthol AS-D chloracetate, the myeloperoxidase (MPO) assay kit and all other chemicals used in this study were purchased from Sigma Aldrich (St Louis, Missouri, USA). Oil-red-O was from Amresco (Solon, Ohio, USA); Hep3B and HepG2 cells were purchased from ATCC (Manassas, Virginia, USA). Cell culture media were from Gibco Life Technologies (Grand Island, New York, USA).

### Animal model of alcoholic steatohepatitis

**Figure 1:**
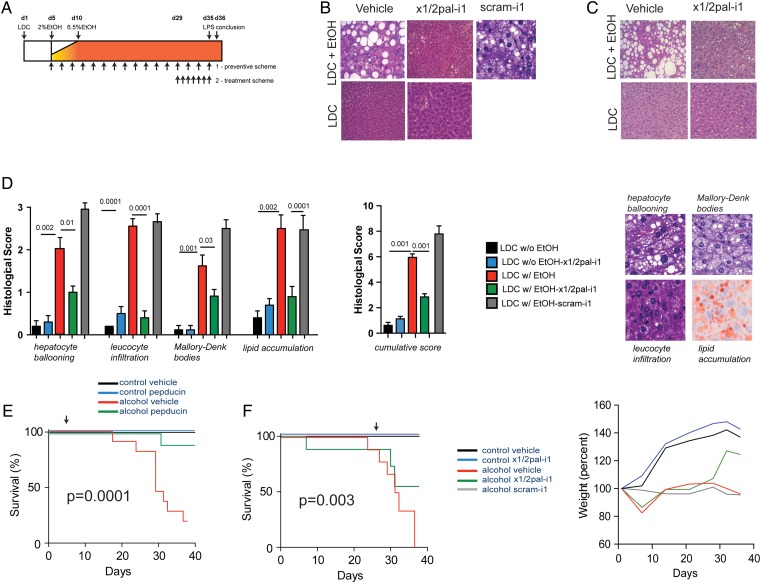
x1/2pal-i1 treatment inhibited the development and progression of alcoholic liver disease. (A) Experimental approach. Mice received the Lieber–DeCarli (LDC) diet. Pepducin therapy was commenced either with the introduction of ethanol (day 5; ‘preventative setting’; 2.5 mg/kg x1/2pal-i1 subcutaneous every other day) or after mice had established disease (day 29, ‘therapeutic setting’; 5.0 mg/kg x1/2pal-i1 subcutaneous once a day). On day 35, mice received LPS (2.5 mg/kg intraperitoneal) and were assessed 24 h later. (B) x1/2pal-i1 prevents development of liver steatosis. Representative liver sections stained with H&E (n=15). (C) Therapeutic x1/2pal-i1 reverts liver steatosis. Representative liver sections stained with H&E (n=15). (D) Histological disease activity. H&E-stained sections for hepatocyte ballooning, leucocyte infiltration and Mallory–Denk bodies, Oil-red-O for steatosis. Statistical analysis: Mann–Whitney U after Kruskal–Wallis; n=15 per group; *p<0.05 (LDC vs LDC-EtOH); †p<0.05 (LDC-EtOH vs LDC-EtOH-x1/2pal-i1). (E) x1/2pal-i1 treatment prevents from alcohol-induced mortality. Mice received 2.5 mg/kg of x1/2pal-i1, scram-i1 (2.5 mg/kg) or vehicle control every other day. Statistical analysis: Mantel–Cox test p<0.0001. n=15. (F) x1/2pal-i1 therapy reduces alcohol-induced mortality and prevents from weight loss. Mice received 5 mg/kg of x1/2pal-i1, scram-i1 or vehicle control every day from day 29 until the conclusion of the experiment. The animals’ weight was taken every other day and weight curves were compared. Statistical analysis: Mantel–Cox test p<0.0001. n=15 (n=7 for scram-i1).

Mouse protocols were approved by the relevant authorities and all procedures were performed in accordance with the institutional guidelines. Also, 5–6-week-old female C57Bl/6 mice were purchased from Charles River Laboratories. The animals were housed under specific pathogen-free conditions. Animals were allowed to acclimatise for 7–10 days before the start of the experiments. Mice were subjected to a high-fat LDC (44% fat-derived, 16% protein-derived and 40% carbohydrate or ethanol-derived calories).^32^ Ethanol was introduced after 5 days, starting 2% (v/v) ethanol, and was then increased every other day to a final concentration of 6.5% (v/v) ethanol on the 10th day of the experiment. Control animals received LDC without any alcohol.^32^ As outlined in figure 1, we started to inject the x1/2pal-i1 pepducin (2.5 mg/kg, every other day) either concomitantly with the introduction of ethanol (day 5) in a preventative mode or after mice had been on alcohol containing LDC for 4 weeks. The latter therapeutically treated mice were injected with x1/2pal-i1 (5 mg/kg) every day for the remaining 7 days of the experiment. The vehicle (10% dimethyl sulfoxide (DMSO)) and was injected as a control. Mice received one single low-dose injection of endotoxin (2.5 mg/kg) intraperitoneally 24 h before sacrificing.^32^ Human alcoholic steatohepatitis (ASH) is characterised by high blood endotoxin levels, which cannot be observed in the murine model. Upon completion of the study, the animals’ weight was taken before terminal anaesthesia with ketamine/xylazine. Cardiac punctures were performed to collect blood. Livers were weighed and either embedded in paraffin, frozen or prepared for RNA extraction, triglyceride extraction or MPO assessment.

### Measurement of serum and liver cytokine levels

Serum CXCL8 levels were measured by ELISA. Liver cytokine mRNA levels were measured by real-time quantitative SYBR-green PCR. Primers were designed as follows: tumour necrosis factor (TNF)α: 5′-tgggagtagacaaggtacaaccc-3′ (forward) and 5′-catcttctcaaaattcgagtgacaa-3′ (reverse). CXCL1: 5′-ctgggattcacctcaagaacatc-3′ (forward) and 5′-cagggtcaaggcaagcctc-3′ (reverse), IL-1β 5′-tgaaaacacagaagtaacgtccg-3′(forward) and 5′-cccaggaggaaattgtaatggga-3′.

MPO was extracted from liver tissue and measured by ELISA. Aspartate aminotransferase (AST) and alanine aminotransferase (ALT) were measured by a colorimetric enzyme activity assay (Sigma Aldrich).

### Histological assessments of murine liver tissue

Liver sections were embedded in paraffin and cut into 5–15-µm-thick slices by subsequent staining with H&E. Liver morphology was then assessed microscopically. To compare the numbers of neutrophils in the livers of pepducin-treated versus vehicle-treated animals, paraffin was removed and slides were stained with naphthol AS-D chloroacetate (Sigma Aldrich).^33^ Neutrophil numbers were counted in four representative microscopic fields from 10 individual mice per group.

For the assessment of liver tissue lipid content, frozen sections were stained with Oil-red-O (Sigma Aldrich). Accumulated lipid appears as bright red droplets in the liver tissue and the amount of these bright red areas was then compared microscopically.

### Measurement of liver triglycerides from liver tissue

The lipid liver fraction was extracted by the Folch method.^34^ Briefly, 100 mg of liver tissue were homogenised in the presence of 3:1 chloroform:methanol. After filtration and two washing steps, the lipid extracts were analysed by a photometric assay (Roche/Hitachi).

### Immunoblotting of caspase-1

Snap frozen liver samples were homogenised and separated by 12% sodium dodecyl sulfate polyacrylamide gel electrophoresis. After blotting, membranes were probed by a polyclonal anti-caspase-1 p20 antibody (Santa Cruz, clone M19).

### In vitro neutrophil chemotaxis

Human neutrophils were obtained from EDTA anticoagulated blood of healthy volunteers. After density gradient centrifugation, remaining red blood cells were lysed. Cell preparations typically yielded >95% neutrophils with almost 100% viability.

Neutrophil migration was measured using a 48-well micro chemotaxis chamber (Neuroprobe, Gaithersburg, Maryland, USA). Cells were allowed to migrate for 30 min into a 5 µm pore-sized nitrocellulose membrane and were stained subsequently. Migration depth was quantified microscopically by evaluating the difference between cells that did not migrate and the leading front of neutrophils.

### Cell culture

Hep3b cells were cultured in Dulbecco's modified Eagle's medium (DMEM) cell culture media containing 10% fetal bovine serum (FBS). HepG2 cells were cultured in Roswell Park Memorial Institute medium containing 10% FBS. For some experiments, Hep3b cells were stimulated with ethanol or TNFα. IL-8 levels were measured in the supernatants of Hep3b cells and subjected to chemotaxis assays. To indicate generalisability, some of the experiments were repeated in HepG2 cells. To assess potential cytotoxicity of pepducins on hepatocytes, we performed MTT tests. Hep3B cells were incubated with DMEM/0.2% FBS for 48 h in 96-well plates. Thereafter, cells were incubated with different concentrations of the x1/2pal-i1 pepducin, control medium or doxorubicin IC_50_ (300 nM) for another 48 h. MTT (10 µL/well) was added and incubated for another 5 h. After the addition of 100 µL DMSO, the extinction was measured photometrically (wavelength 550 nm).

## Results

### High-fat diet supplemented with alcohol induces experimental ASH

**Figure 2:**
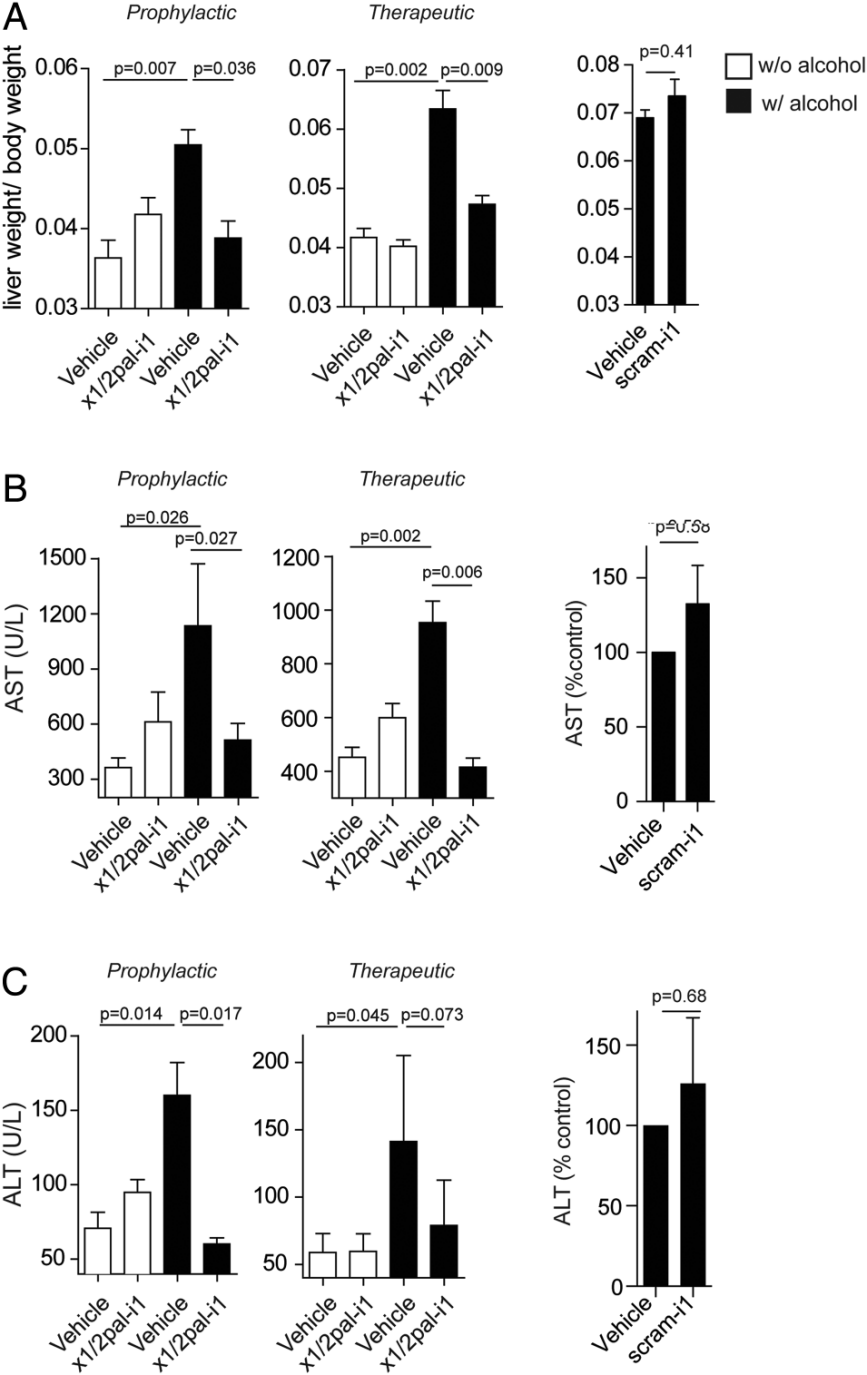
x1/2pal-i1 treatment normalises liver–body weight ratio and prevents from hepatic necrosis. (A) Prophylactic and therapeutic x1/2pal-i1 administration result in a normal liver–body weight ration. Statistical analysis: Mann–Whitney U after Kruskal–Wallis. n=15 per group (n=7 for scram-i1 experiment). (B and C) Prophylactic and therapeutic x1/2pal-i1 treatment prevents from liver cell necrosis. Aspartate aminotransferase (AST) and alanine aminotransferase (ALT) levels as markers of liver cell necrosis. Statistical analysis: Mann–Whitney U after Kruskal–Wallis. n=15 per group (n=7 for scram-i1 experiment).

We administered C57Bl/6 mice with an LDC high-fat diet,^32^ supplemented with ethanol, followed by intraperitoneal lipopolysaccharide (LPS) injection 24 h prior to sacrifice as detailed in figure 1A. H&E stainings of liver sections from mice receiving an ethanol-enriched LDC diet (LDC^EtOH^), but not LDC diet alone (LDC^Ctrl^), resulted in severe steatohepatitis, characterised by neutrophilic infiltration and hepatocyte ballooning, lipid accumulation and Mallory–Denk bodies (figure 1B–D). While microvesicular steatosis was observed in most of the liver sections of LDC^Ctrl^ mice (figure 1B, C), addition of alcohol resulted in a macrovesicular pattern with hepatocyte ballooning in LDC^EtOH^ mice (figure 1B, C). These features (figure 1D) closely resemble the characteristic histological picture observed in human ASH.^35^ LDC^EtOH^ mice exhibited significant mortality and developed cachexia (figure 1E, F) over the course of the experiment, even prior to LPS administration (figure 1F). At the end of the experiment, the liver-to-body weight ratio in LDC^EtOH^ mice was more than twofold higher compared with LDC^Ctrl^ mice (figure 2A, B), which was associated with profoundly increased AST and ALT serum levels, reflecting increased hepatocyte injury (figure 2A, B). Altogether, this experimental model faithfully recapitulates many critical features of human ASH.

### CXCR1/2-specific antagonist pepducin x1/2pal-i1 prevents steatohepatitis and mortality in experimental ASH

With hepatic neutrophil infiltration being a key characteristic and IL-8 levels correlated with outcome of human ASH,^17^ ^18^ we asked whether CXCR1/2 signalling is causally involved in experimental ASH. We chose a preventative regimen for blocking CXCR1/2 via the x1/2pal-i1 pepducin,^25^ administered at a dose of 2.5 mg/kg every other day, commenced with the introduction of ethanol (figure 1A). To rule out non-specific pepducin effects, we treated one group of mice with a scrambled nonsense pepducin (scram-i1) at a dose of 2.5 mg/kg every other day. Figure 1B demonstrates that x1/2pal-i1 almost completely abrogated histological steatohepatitis in LDC^EtOH^ mice, whereas scram-i1 failed to do so. Importantly, x1/2pal-i1 almost completely protected from mortality in LDC^EtOH^ mice (figure 1E). The profound effect of x1/2pal-i1 was mirrored by normalisation of elevated AST and ALT serum levels in LDC^EtOH^ mice (figure 2B, C), alongside normalisation of liver-to-body weight ratios (figure 2A). Pepducin x1/2pal-i1 did not affect liver histology in LDC^Ctrl^ mice (figure 1B). The scram-i1 pepducin failed to protect mice from liver damage as observed by high AST and ALT levels (figure 2B, C). Additionally, we observed no difference in the liver-to-body weight ratios between LDC^EtOH^ mice treated with DMSO- or scram-i1 injections (figure 2A).

### x1/2pal-i1 is effective in reversing established experimental steatohepatitis

We next investigated whether CXCR1/2 blockade would also be effective therapeutically in fully raging disease. On day 1, we pre-assigned mice to groups to receive either vehicle or x1/2pal-i1, but postponed the start of treatment (daily 5 mg/kg x1/2pal-i1) to day 29 (figure 1A), when LDC^EtOH^ mice had already developed cachexia and significant mortality compared with LDC^Ctrl^ mice (figure 1F). This reflected severe disease present at the start of pepducin treatment. In contrast to vehicle treatment, x1/2pal-i1 indeed reversed histological steatohepatitis in LDC^EtOH^ mice (figure 1C). Liver leucocyte infiltration was entirely prevented by x1/2pal-i1 in LDC^EtOH^ mice, remarkably with almost complete prevention of pathological lipid accumulation in the liver (figure 1D). Similarly, elevated AST and ALT serum levels in vehicle-treated mice returned to baseline upon treatment with x1/2pal-i1 (figure 2B, C). Impressively, while the group pre-assigned to receive x1/2pal-i1 from day 29 onwards trended towards more severe disease prior to pepducin therapy (figure 1F), treatment with x1/2pal-i1 reversed this trend and resulted in a statistically significantly better overall survival at the end of the experiment compared with vehicle-treated LDC^EtOH^ mice (figure 1F). This beneficial effect of therapeutic x1/2pal-i1 was mirrored by almost complete normalisation of liver-to-body weight ratios in LDC^EtOH^ mice (figure 2A), alongside a steep increase towards normalisation of total body weight after institution of x1/2pal-i1 treatment (figure 1F). Altogether, these data establish that CXCR1/2 signalling is a critical driver of experimental ASH and that therapeutic intervention in established disease via the specific pepducin x1/2pal-i1 reverses disease and prevents mortality.

### x1/2pal-i1 treatment abrogates pro-inflammatory cytokine transcription and downregulates caspase-1 expression

**Figure 3:**
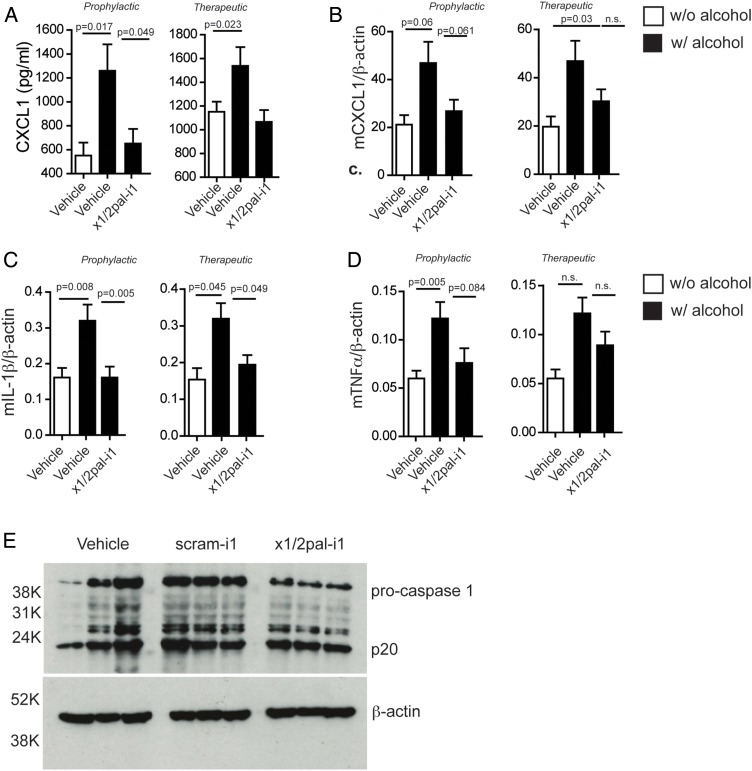
CXCR2 blockade reduces pro-inflammatory cytokine expression. Mice on Lieber–DeCarli diet were injected with x1/2pal-i1 in a prophylactic or therapeutic mode. (A) x1/2pal-i1 pepducin treatment decreases serum CXCL1. CXCL1 serum levels were measured by ELISA. (B) CXCR1/2 blockade by the x1/2pal-i1 pepducin decreases hepatic CXCL1 mRNA transcription. mRNA levels were measured by SYBR-green real-time PCR. (C) CXCR1/2 blockade by the x1/2pal-i1 pepducin decreases hepatic interleukin (IL)-1β mRNA transcription. mRNA levels were measured by SYBR-green real-time PCR (D) CXCR1/2 blockade by the x1/2pal-i1 pepducin decreases hepatic tumour necrosis factor (TNF)α mRNA transcription. mRNA levels were measured by SYBR-green real-time PCR. Statistical analysis: Mann–Whitney U after Kruskal–Wallis. n=6 (E) CXCR1/2 blockade reduces pro-caspase-1 expression in livers. Liver tissue lysates were immunoblotted for caspase-1 protein expression. Representative western blot analysis, n=3, each lane represents an individual liver specimen.

Human ASH is accompanied by high serum levels of IL-8 and increased hepatic expression of IL-8, TNFα and IL-1β.^15^ ^17^ ^18^ LDC^EtOH^ mice exhibited increased serum levels of CXCL1 compared with LDC^Ctrl^ mice (figure 3A). Hepatic mRNA expression of *Cxcl1*, *Tnf* and *Il1β* was increased in LDC^EtOH^ compared with LDC^Ctrl^ mice (figure 3B–D). Increased *Il1β* expression in LDC^EtOH^ mice was prevented by x1/2pal-i1 administration (figure 3C), and even when administration was delayed until disease had established (figure 3C). CXCL1 serum levels were similarly reduced (figure 3A) by x1/2pal-i1, while *Cxcl1* and *Tnf* mRNA levels only trended lower (figure 3B, C). These data show that x1/2pal-i1 treatment of experimental ASH normalises hepatic expression of inflammatory cytokines, which are characteristically induced in human ASH.

Patients suffering from ASH exhibit increased caspase-1 expression.^36^ ^37^ Similarly, chronic ethanol exposure in mice induces pro-caspase-1 expression.^37^ To test whether blocking CXCR2 receptors by x1/2pal-i1 decreases inflammasome activation, we probed liver tissue lysates for caspase-1, a hallmark of inflammasome activity. Our results indicate that the blockade of CXCR2 reduces the amount of pro-caspase-1 in x1/2pal-i1-treated mice, whereas DMSO and scram-1 treatment had no effect. Interestingly, no differences in the amount of active caspase-1 (p20) were detected, which could, however, be a result of a relatively short half-life of cleaved caspase-1 (figure 3E).^37^ ^38^

### x1/2pal-i1 pepducin abrogates neutrophil accumulation in experimental ASH

**Figure 4:**
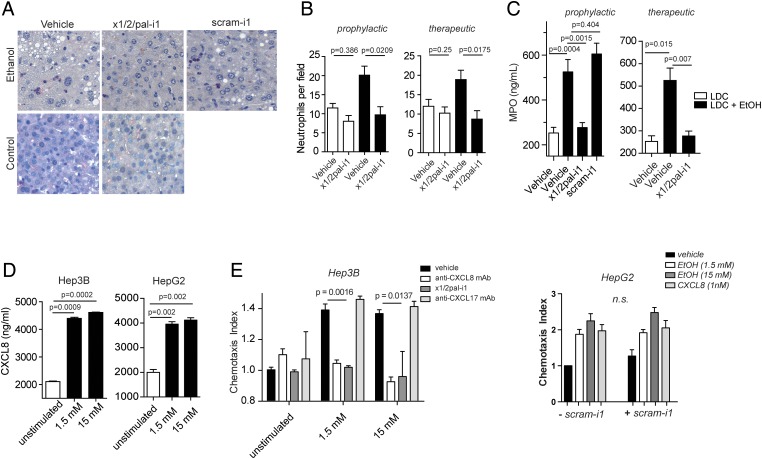
Pepducin treatment inhibits neutrophil infiltration and activation. (A and B) x1/2pal-i1 prevents accumulation of neutrophils in liver tissue sections of alcoholic mice. To compare the numbers of neutrophils, histological sections were stained with naphthol AS-D chloroacetate. Neutrophil numbers were counted in four representative microscopic fields from 10 individual mice per group. (C) x1/2pal-i1 treatment reduces myeloperoxidase (MPO) levels in liver tissue. Homogenised liver tissue was assessed for MPO levels. Right panel: preventative treatment; left panel: therapeutic treatment. Statistical analysis: Mann–Whitney U after Kruskal–Wallis. n=10. (D) Ethanol induces CXCL1 secretion in Hep3B and HepG2 cells. CXCL1 levels were measured after 24 h of ethanol exposure to the cells. Mann–Whitney U after Kruskal–Wallis, n=3 (E) Ethanol-induced neutrophil chemotaxis is CXCL8 dependent. CXCL8 (IL-8) was neutralised by adding antibodies directed against CXCL8. CXCR1 and CXCR2 receptor signalling was inhibited by x1/2pal-i1. The nonsense pepducin scram-i1 had no effect on neutrophil chemotaxis induced by HepG2 cell supernatants. Chemotaxis experiments were performed in modified Boyden chambers. Statistical analysis: Mann–Whitney U after Kruskal–Wallis, n=3. LDC, Lieber–DeCarli diet.

Hepatic neutrophil accumulation is a characteristic feature of ASH and is similarly observed in experimental ASH (figure 4A, B), as revealed through staining with naphthol AS-D chloroacetate, a chromogenic substrate of the specific esterases of neutrophilic granules. Administration of x1/2pal-i1 to LDC^EtOH^ mice reduced the numbers of neutrophils per high-power field to those observed in LDC^Ctrl^ mice (figure 4A). Activated neutrophils have a short half-life and high turnover in inflamed tissue. Delayed CXCR1/2 blockade by x1/2pal-i1 likewise resulted in a similar complete abrogation of neutrophil accumulation in LDC^EtOH^ mice (figure 4B), whereas scram-1 had no effect on neutrophil numbers. Finally, liver MPO levels were decreased to baseline levels in x1/2pal-i1-treated LDC^EtOH^ mice (figure 4C).

### Hepatocyte Hep3b and HepG2 cells secrete CXCL8 upon alcohol exposure, attracting neutrophils in vitro

In addition to liver Kupffer cells, hepatocytes also contribute profoundly to inflammatory cytokine secretion in the liver. We chose the human Hep3b and HepG2 cell lines to test the hypothesis that ethanol exposure directly induces CXCL1 secretion in hepatocytes. As shown in figure 4D, 1.5 and 15 mM ethanol in culture medium resulted in a substantial increase in CXCL8 release from Hep3b and HepG2 cells. Supernatants from ethanol-exposed Hep3b and HepG2 (data not shown) cells exhibited chemotactic activity to neutrophils in vitro, and this activity was completely blocked either by a neutralising anti-CXCL8 monoclonal antibody and x1/2pal-i1, whereas a blocking anti-CXCL17 antibody had no effect (figure 4E). In another set of experiments, human neutrophil chemotaxis towards supernatants of ethanol-treated HepG2 cells was performed in the presence of scram-1. Scram-1 had no effect on the induction of chemotaxis towards the supernatants. CXCL8 (1 nM) served as control (figure 4E). Altogether, these data suggest that upon ethanol-induced tissue damage in experimental ASH, injured hepatocytes may release ligands for CXCR1/2, which recruit neutrophils into the liver, which in turn may release cytotoxic contents of their granula, resulting in the characteristic tissue damage of ASH.

### x1/2pal-i1 treatment prevents lipid accumulation in experimental ASH

**Figure 5:**
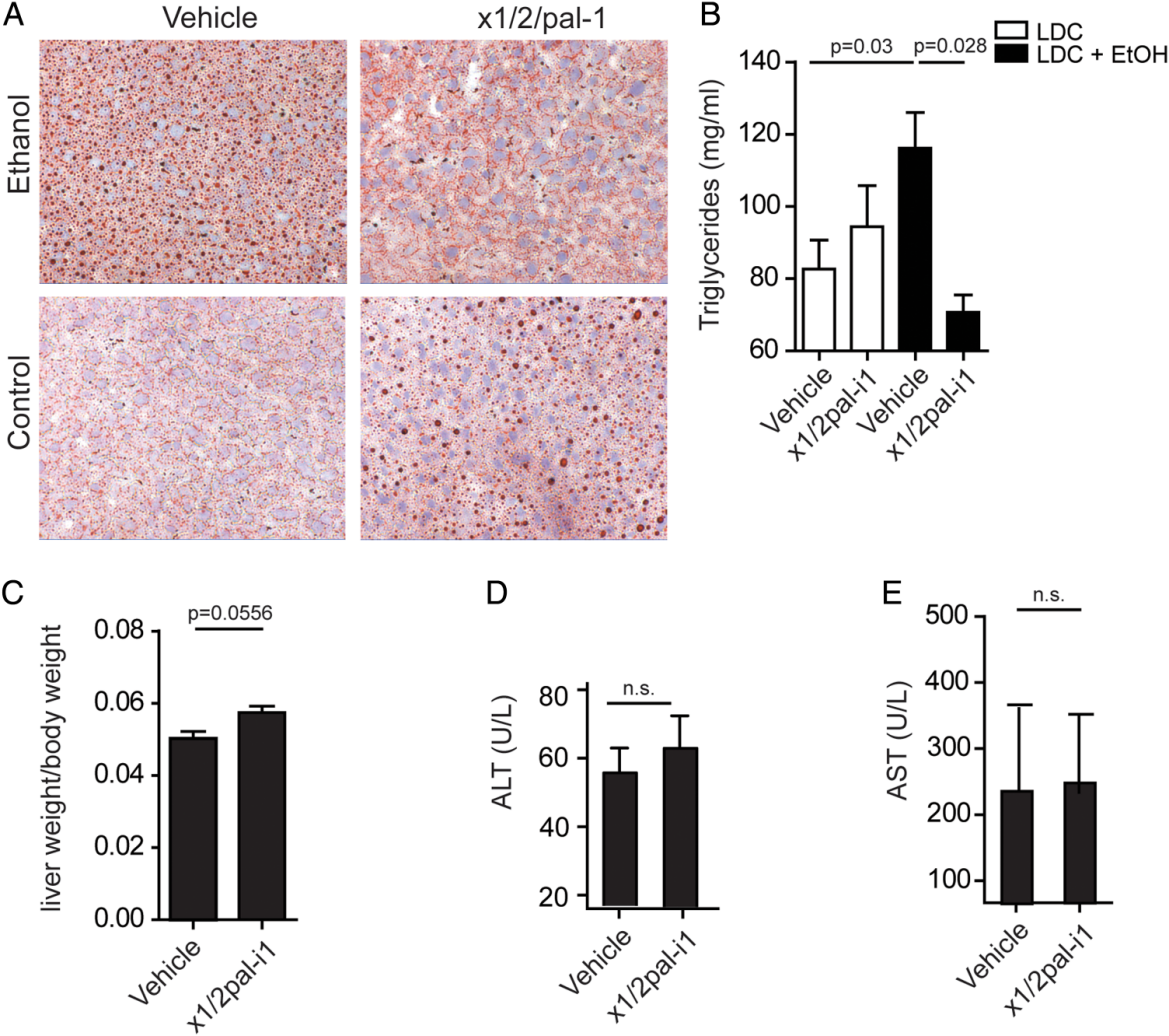
(A) CXCR1/2 pepducin blockade reverses steatosis in alcoholic mice. Oil-red-O stain of liver sections of mice treated therapeutically. N=15. (B) Blockade of CXCR2 receptors decreases the triglyceride content in livers. Triglycerides from liver tissue of mice treated therapeutically were measured by a photometric assay. N=10. Statistical analysis: Mann–Whitney U test after Kruskal–Wallis. (C–E) The x1/2pal-i1 pepducin has no effect in mice on regular chow diet. Mice received the animal facility's standard chow diet. The animals were injected with either 10% of dimethyl sulfoxide or the x1/2pal-i1 pepducin (5 mg/kg) subcutaneous every other day. After 5 weeks of treatment, the animals were sacrificed and evaluated for (C) liver–body weight ratio, (D) alanine aminotransferase (ALT) and (E) aspartate aminotransferase (AST). N=10. Statistical analysis: Mann–Whitney U after Kruskal–Wallis; n.s.: p>0.05. LDC, Lieber–DeCarli diet.

Hepatocyte lipid accumulation is a cardinal feature of ASH.^3^ LDC^Ctrl^ mice exhibited little fat accumulation in their livers as determined by Oil-red-O staining, whereas the number of hepatic lipid vesicles was drastically increased in LDC^EtOH^ mice (figure 5A). Administration of x1/2pal-i1 completely prevented this increase in lipid vesicles in LDC^EtOH^ mice (figure 5A). Triglycerides are thought to represent the primary lipid species in ballooned hepatocytes in ASH.^39^ We, therefore, quantified triglycerides within the lipophilic fraction extracted from whole murine livers via the Folch method. Triglyceride content in LDC^EtOH^ mice was significantly increased compared with LDC^Ctrl^ mice (figure 5B). x1/2pal-i1 completely prevented this increase in LDC^EtOH^ mice to levels below those observed in LDC^Ctrl^ mice (figure 5B). Compared with vehicle-treated LDC^Ctrl^ mice, both Oil-red-O^+^ lipid vesicles and liver triglyceride content trended higher upon x1,2pal-i1 treatment (figure 5A, B). However, this was not associated with increased liver/body weight ratio (figure 2A). To exclude potential toxicity of x1/2pal-i1, higher doses (5 mg/kg, every other day) were administered in mice on a normal diet. We did not observe an increase in ALT (figure 5D) or AST (figure 5E) levels in serum nor was the liver-to-body weight ratio altered compared with vehicle-treated mice (figure 5C).

## Conclusion

**Figure 6:**
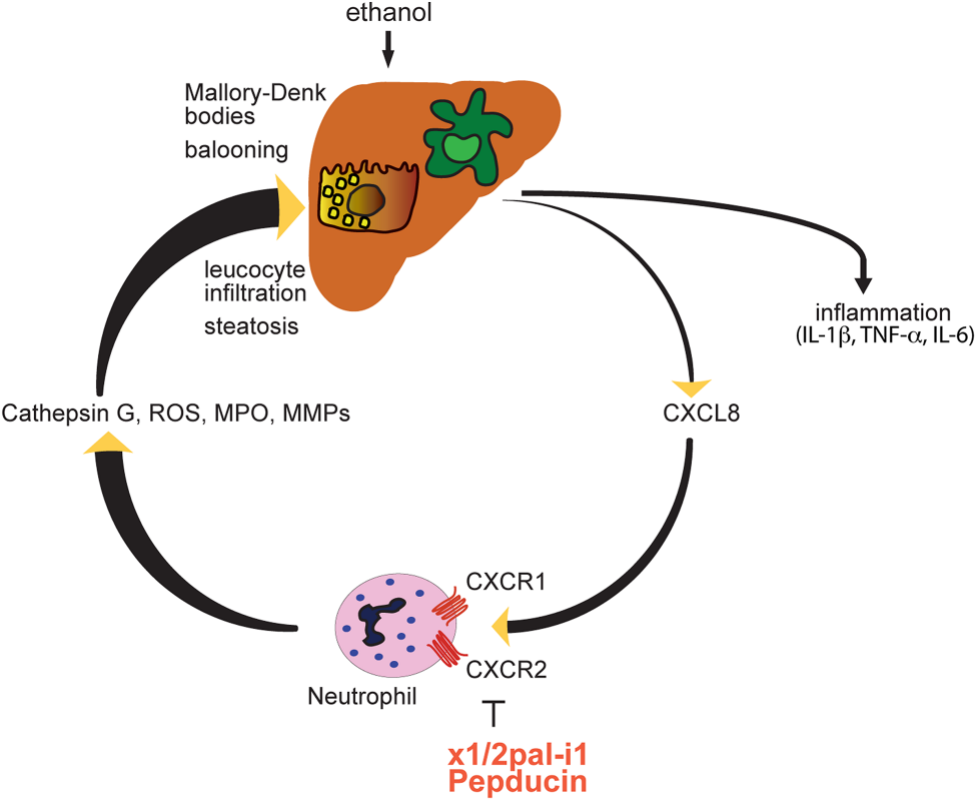
Proposed mechanism of x1/2pal-i1 therapy. IL, interleukin; MMP, matrix metalloproteinases; MPO, myeloperoxidase; ROS, reactive oxygen species; TNF, tumour necrosis factor.

Here, we demonstrate that CXCR1/2 signalling plays a central role in an experimental model of ASH, which phenocopies core features of human ASH. Remarkably, late therapeutic blockade of CXCR1/2 signalling with the pepducin x1/2pal-i1 in established, severe disease, was effective in preventing further mortality and reversing liver inflammation (figure 6).

Neutrophil infiltration in the liver is a classic hallmark of human ASH, and correspondingly observed in experimental ASH. Efficacy of x1/2pal-i1 in experimental ASH was associated with a profound reduction of neutrophil granulocytes infiltrating the liver, signifying a critical role of neutrophils in this disease. More specifically, neutrophils are recruited to sites of invading pathogens or tissue injury within minutes.^40^ Apart from removing pathogens by phagocytosis, neutrophils, however, release an array of mediators such as MPO, matrix metalloproteases, serine proteases, elastases, cathepsin G and nicotinamide adenine dinucleotide phosphate oxidase, which can promote tissue damage.^41^ Hepatic neutrophil infiltration has also been demonstrated in other experimental models of liver disease such as ischaemia-reperfusion,^42^ ^43^ ConA-induced and CCl_4_-induced hepatitis.^44^ Interestingly, in a murine sepsis model we have previously shown that CXCR1/2 blockade results in suppression of the systemic inflammatory response syndrome without yielding higher bacterial loads.^25^ Blocking CXCR1 and CXCR2 receptors with pepducins did not suppress chemotaxis induced by other neutrophil chemoattractants; therefore, x1/2pal-i1 may be rather considered immunomodulatory rather than immunosuppressive.^25^ Blocking neutrophil emigration from the blood stream into liver tissue and preventing their overzealous activation via x1/2pal-i1 may represent an intervention at a critical checkpoint that shuts off organ destruction, resulting in protection from acute liver failure, and may also prevent further consequences such as fibrosis.^45^

CXCR1/2 blockade via x1/2pal-i1 was associated with normalisation of increased hepatic IL-1β expression during experimental ASH. IL-1β secretion due to caspase-1 activation in Kupffer cells has previously been shown to induce hepatic steatosis, and blockade with recombinant IL-1 receptor antagonist (IL-1Ra, Anakinra) in experimental steatohepatitis attenuated liver inflammation.^37^ In our model of experimental ASH, we show that blocking CXCR2 receptors downregulates the expression of pro-caspase 1. Interestingly, Marques *et al*^46^ reported that DF2156a, an allosteric small peptide inhibitor of CXCR1/2, reduced the number of infiltrating neutrophils in paracetamol-induced liver injury, but this was not associated with a reduction in liver injury. The latter required co-blockade of the formyl peptide receptor 1,^46^ which could either point to differences in pathogenesis of these liver injury models or the specific potency of x1/2-pal-i1 in blocking CXCR1/2 signalling.

The IL-8 receptors CXCR1 and CXCR2 are not only expressed on leucocytes but can also be found on human hepatocytes under pathological conditions.^42^ ^43^ Upon ischaemia/reperfusion injury, CXCR1 is upregulated on murine hepatocytes and appears involved in hepatocyte proliferation. Genetic deletion of CXCR1 or its blockade by repertaxin resulted in a significant decrease of BrdU incorporation into proliferating hepatocytes.^47^ In this model of ischaemia/reperfusion injury, which indeed causes one single assault to the liver tissue, immigrating neutrophils are a good prognostic factor and facilitate repair and regeneration.^47^ In ASH, in contrast, with alcohol present as a constant noxa, repair and regeneration might result in hyperproliferation and consequent development of neoplasia. Liver cirrhosis bears a high risk for developing hepatocellular carcinoma, and it might be speculated that increased hepatocyte CXCR1 expression consequent to liver injury could promote pathological hyperproliferation. Indeed, CXCL5, another CXCR2 ligand, has been shown to be associated with a high neutrophil load and poor outcome in hepatocellular carcinoma.^48^ CXCR1 and CXCR2 also promote neoangiogenesis in tumour tissue.^30^ ^31^ Hence, blockade of CXCR1/2 signalling may not only beneficially affect acute inflammation and the development of steatosis, but could potentially also decrease the risk of hepatocellular carcinoma.

Treatment for severe ASH includes supportive measures to reduce ascites, prevent infections, treat hepatic encephalopathy (antibiotics, lactulose), ascertain sufficient protein intake and maintain serum albumin levels, and supplementation of vitamins (thiamine).^2^ The use of corticosteroids in ASH remains controversial due to the increased infection risk,^49^ while some lines of evidence suggest that pentoxifylline may be associated with improved in-hospital survival.^50^ Targeting TNFα has yielded mixed results in clinical trials, complicated by increased risk of severe infections and associated with increased mortality rates, and is therefore currently not recommended.^51–53^ The only treatment that has shown to significantly improve the long-term outcome of AH is liver transplantation.^54^ The treatment of patients with acute alcoholic liver disease with CXCR1/2 blocking pepducins, therefore, deserves evaluation in clinical trials. The first clinical phase 1 trial with a pepducin inhibitor of protease-activated receptor 1^55^ has been successfully completed (http://clinicaltrials.gov/ct2/show/NCT01806077), potentially paving the way for clinical development of other pepducins such as the CXCR1/2-directed x1/2pal-i1 pepducin for severe ASH.

## References

[1] Mokdad AH, Marks JS, Stroup DF, et al Actual causes of death in the United States, 2000. JAMA 2004;291:1238–45. 10.1001/jama.291.10.1238 15010446

[2] Lucey MR, Mathurin P, Morgan TR Alcoholic hepatitis. N Engl J Med 2009;360:2758–69. 10.1056/NEJMra0805786 19553649

[3] Gao B, Bataller R Alcoholic liver disease: pathogenesis and new therapeutic targets. Gastroenterology 2011;141:1572–85. 10.1053/j.gastro.2011.09.002 21920463 PMC3214974

[4] Ramaiah SK, Jaeschke H Hepatic neutrophil infiltration in the pathogenesis of alcohol-induced liver injury. Toxicol Mech Methods 2007;17:431–40. 10.1080/00952990701407702 20020946

[5] Gao B, Xu M Chemokines and alcoholic hepatitis: are chemokines good therapeutic targets? Gut 2014;63:1683–4. 10.1136/gutjnl-2013-306603 24515805 PMC5451264

[6] Mathurin P, O'Grady J, Carithers RL, et al Corticosteroids improve short-term survival in patients with severe alcoholic hepatitis: meta-analysis of individual patient data. Gut 2011;60:255–60. 10.1136/gut.2010.224097 20940288

[7] Dunn W, Jamil LH, Brown LS, et al MELD accurately predicts mortality in patients with alcoholic hepatitis. Hepatology 2005;41:353–8. 10.1002/hep.20503 15660383

[8] Kubes P, Mehal WZ Sterile inflammation in the liver. Gastroenterology 2012;143:1158–72. 10.1053/j.gastro.2012.09.008 22982943

[9] Setshedi M, Wands JR, Monte SM Acetaldehyde adducts in alcoholic liver disease. Oxid Med Cell Longev 2010;3:178–85. 10.4161/oxim.3.3.12288 20716942 PMC2952076

[10] Kwon HJ, Won YS, Park O, et al Aldehyde dehydrogenase 2 deficiency ameliorates alcoholic fatty liver but worsens liver inflammation and fibrosis in mice. Hepatology 2014;60:146–57. 10.1002/hep.27036 24492981 PMC4077916

[11] Keshavarzian A, Farhadi A, Forsyth CB, et al Evidence that chronic alcohol exposure promotes intestinal oxidative stress, intestinal hyperpermeability and endotoxemia prior to development of alcoholic steatohepatitis in rats. J Hepatol 2009;50:538–47. 10.1016/j.jhep.2008.10.028 19155080 PMC2680133

[12] Hritz I, Mandrekar P, Velayudham A, et al The critical role of toll-like receptor (TLR) 4 in alcoholic liver disease is independent of the common TLR adapter MyD88. Hepatology 2008;48:1224–31. 10.1002/hep.22470 18792393 PMC7137387

[13] Keshavarzian A, Fields J Alcoholic liver disease: is it an “extraintestinal” complication of alcohol-induced intestinal injury? J Lab Clin Med 2003;142:285–7. 10.1016/S0022-2143(03)00140-9 14647031

[14] Wang HJ, Gao B, Zakhari S, et al Inflammation in alcoholic liver disease. Annu Rev Nutr 2012;32:343–68. 10.1146/annurev-nutr-072610-145138 22524187 PMC3670145

[15] Dominguez M, Miquel R, Colmenero J, et al Hepatic expression of CXC chemokines predicts portal hypertension and survival in patients with alcoholic hepatitis. Gastroenterology 2009;136:1639–50. 10.1053/j.gastro.2009.01.056 19208360

[16] Affò S, Dominguez M, Lozano JJ, et al Transcriptome analysis identifies TNF superfamily receptors as potential therapeutic targets in alcoholic hepatitis. Gut 2013;62:452–60. 10.1136/gutjnl-2011-301146 22637703 PMC4064940

[17] Hill DB, Marsano LS, McClain CJ Increased plasma interleukin-8 concentrations in alcoholic hepatitis. Hepatology 1993;18:576–80. 10.1002/hep.1840180316 8359798

[18] Huang YS, Chan CY, Wu JC, et al Serum levels of interleukin-8 in alcoholic liver disease: relationship with disease stage, biochemical parameters and survival. J Hepatol 1996;24:377–84. 10.1016/S0168-8278(96)80156-5 8738722

[19] Muller WA Leukocyte-endothelial-cell interactions in leukocyte transmigration and the inflammatory response. Trends Immunol 2003;24:326–33. 10.1016/S1471-4906(03)00117-0 12810109

[20] Griffith JW, Sokol CL, Luster AD Chemokines and chemokine receptors: positioning cells for host defense and immunity. Annu Rev Immunol 2014;32:659–702. 10.1146/annurev-immunol-032713-120145 24655300

[21] Venkatakrishnan AJ, Deupi X, Lebon G, et al Molecular signatures of G-protein-coupled receptors. Nature 2013;494:185–94. 10.1038/nature11896 23407534

[22] Kaneider NC, Leger AJ, Kuliopulos A Therapeutic targeting of molecules involved in leukocyte-endothelial cell interactions. FEBS J 2006;273:4416–24. 10.1111/j.1742-4658.2006.05441.x 16956369

[23] Covic L, Gresser AL, Talavera J, et al Activation and inhibition of G protein-coupled receptors by cell-penetrating membrane-tethered peptides. Proc Natl Acad Sci USA 2002;99:643–8. 10.1073/pnas.022460899 11805322 PMC117359

[24] Covic L, Misra M, Badar J, et al Pepducin-based intervention of thrombin-receptor signaling and systemic platelet activation. Nat Med 2002;8:1161–5. 10.1038/nm760 12357249

[25] Kaneider NC, Agarwal A, Leger AJ, et al Reversing systemic inflammatory response syndrome with chemokine receptor pepducins. Nat Med 2005;11:661–5. 10.1038/nm1245 15880119

[26] Kaneider NC, Leger AJ, Agarwal A, et al ‘Role reversal’ for the receptor PAR1 in sepsis-induced vascular damage. Nat Immunol 2007;8:1303–12. 10.1038/ni1525 17965715 PMC3059149

[27] Tsuji M, Ueda S, Hirayama T, et al FRET-based imaging of transbilayer movement of pepducin in living cells by novel intracellular bioreductively activatable fluorescent probes. Org Biomol Chem 2013;11:3030–7. 10.1039/c3ob27445d 23532512

[28] Leger AJ, Jacques SL, Badar J, et al Blocking the protease-activated receptor 1–4 heterodimer in platelet-mediated thrombosis. Circulation 2006;113:1244–54. 10.1161/CIRCULATIONAHA.105.587758 16505172

[29] O'Callaghan K, Lee L, Nguyen N, et al Targeting CXCR4 with cell-penetrating pepducins in lymphoma and lymphocytic leukemia. Blood 2012;119:1717–25. 10.1182/blood-2011-04-347518 22186993 PMC3286348

[30] Agarwal A, Tressel SL, Kaimal R, et al Identification of a metalloprotease-chemokine signaling system in the ovarian cancer microenvironment: implications for antiangiogenic therapy. Cancer Res 2010;70:5880–90. 10.1158/0008-5472.CAN-09-4341 20570895 PMC2917243

[31] Jamieson T, Clarke M, Steele CW, et al Inhibition of CXCR2 profoundly suppresses inflammation-driven and spontaneous tumorigenesis. J Clin Invest 2012;122:3127–44. 10.1172/JCI61067 22922255 PMC3428079

[32] Lieber CS, DeCarli LM The feeding of alcohol in liquid diets: two decades of applications and 1982 update. Alcohol Clin Exp Res 1982;6:523–31. 10.1111/j.1530-0277.1982.tb05017.x 6758624

[33] Wiedermann CJ, Schmalzl F, Braunsteiner H Investigation of granulocytopoietic kinetics by microdensitometric evaluation of primary granule naphthol-AS-D-chloroacetate esterase activity. Blut 1983;47:271–7. 10.1007/BF00319896 6626750

[34] Folch J, Lees M, Sloane Stanley GH A simple method for the isolation and purification of total lipides from animal tissues. J Biol Chem 1957;226:497–509. 13428781

[35] Altamirano J, Miquel R, Katoonizadeh A, et al A histologic scoring system for prognosis of patients with alcoholic hepatitis. Gastroenterology 2014;146:1231–9. e1236 10.1053/j.gastro.2014.01.018 24440674 PMC3992184

[36] Szabo G, Petrasek J, Bala S Innate immunity and alcoholic liver disease. Dig Dis 2012;30 (Suppl 1):55–60. 10.1159/000341126 PMC6412139 23075869

[37] Petrasek J, Bala S, Csak T, et al IL-1 receptor antagonist ameliorates inflammasome-dependent alcoholic steatohepatitis in mice. J Clin Invest 2012;122:3476–89. 10.1172/JCI60777 22945633 PMC3461900

[38] Walsh JG, Logue SE, Luthi AU, et al Caspase-1 promiscuity is counterbalanced by rapid inactivation of processed enzyme. J Biol Chem 2011;286:32513–24. 10.1074/jbc.M111.225862 21757759 PMC3173193

[39] Ge X, Leung TM, Arriazu E, et al Osteopontin binding to lipopolysaccharide lowers tumor necrosis factor-α and prevents early alcohol-induced liver injury in mice. Hepatology 2014;59:1600–16. 10.1002/hep.26931 24214181 PMC3966944

[40] Köhler A, De Filippo K, Hasenberg M, et al G-CSF-mediated thrombopoietin release triggers neutrophil motility and mobilization from bone marrow via induction of Cxcr2 ligands. Blood 2011;117:4349–57. 10.1182/blood-2010-09-308387 21224471 PMC3087483

[41] Gunzer M Traps and hyper inflammation—new ways that neutrophils promote or hinder survival. Br J Haematol 2014;164:189–99. 10.1111/bjh.12608 24138538

[42] Van Sweringen HL, Sakai N, Quillin RC, et al Roles of hepatocyte and myeloid CXC chemokine receptor-2 in liver recovery and regeneration after ischemia/reperfusion in mice. Hepatology 2013;57:331–8. 10.1002/hep.26049 22961770 PMC3540195

[43] Kuboki S, Shin T, Huber N, et al Hepatocyte signaling through CXC chemokine receptor-2 is detrimental to liver recovery after ischemia/reperfusion in mice. Hepatology 2008;48:1213–23. 10.1002/hep.22471 18688883 PMC2695827

[44] Kwon HJ, Won YS, Park O, et al Opposing effects of prednisolone treatment on T/NKT cell- and hepatotoxin-mediated hepatitis in mice. Hepatology 2014;59:1094–106. 10.1002/hep.26748 24115096 PMC3943761

[45] Zimmermann HW, Seidler S, Gassler N, et al Interleukin-8 is activated in patients with chronic liver diseases and associated with hepatic macrophage accumulation in human liver fibrosis. PLoS ONE 2011;6:e21381 10.1371/journal.pone.0021381 21731723 PMC3120868

[46] Marques PE, Amaral SS, Pires DA, et al Chemokines and mitochondrial products activate neutrophils to amplify organ injury during mouse acute liver failure. Hepatology 2012;56:1971–82. 10.1002/hep.25801 22532075

[47] Clarke C, Kuboki S, Sakai N, et al CXC chemokine receptor-1 is expressed by hepatocytes and regulates liver recovery after hepatic ischemia/reperfusion injury. Hepatology 2011;53:261–71. 10.1002/hep.24028 21254176 PMC3058860

[48] Zhou SL, Dai Z, Zhou ZJ, et al Overexpression of CXCL5 mediates neutrophil infiltration and indicates poor prognosis for hepatocellular carcinoma. Hepatology 2012;56:2242–54. 10.1002/hep.25907 22711685

[49] Nguyen-Khac E, Thevenot T, Piquet MA, et al Glucocorticoids plus N-acetylcysteine in severe alcoholic hepatitis. N Engl J Med 2011;365:1781–9. 10.1056/NEJMoa1101214 22070475

[50] Akriviadis E, Botla R, Briggs W, et al Pentoxifylline improves short-term survival in severe acute alcoholic hepatitis: a double-blind, placebo-controlled trial. Gastroenterology 2000;119:1637–48. 10.1053/gast.2000.20189 11113085

[51] Naveau S, Chollet-Martin S, Dharancy S, et al A double-blind randomized controlled trial of infliximab associated with prednisolone in acute alcoholic hepatitis. Hepatology 2004;39:1390–7. 10.1002/hep.20206 15122768

[52] Boetticher NC, Peine CJ, Kwo P, et al A randomized, double-blinded, placebo-controlled multicenter trial of etanercept in the treatment of alcoholic hepatitis. Gastroenterology 2008;135:1953–60. 10.1053/j.gastro.2008.08.057 18848937 PMC2639749

[53] Mookerjee RP, Tilg H, Williams R, et al Infliximab and alcoholic hepatitis. Hepatology 2004;40:499–500; author reply 500–491 10.1002/hep.20344 15368459

[54] Burra P, Senzolo M, Adam R, et al Elita, Centers ELT. Liver transplantation for alcoholic liver disease in Europe: a study from the ELTR (European Liver Transplant Registry). Am J Transplant Surgeons 2010;10:138–48. 10.1111/j.1600-6143.2009.02869.x 19951276

[55] Zhang P, Gruber A, Kasuda S, et al Suppression of arterial thrombosis without affecting hemostatic parameters with a cell-penetrating PAR1 pepducin. Circulation 2012;126:83–91. 10.1161/CIRCULATIONAHA.112.091918 22705889 PMC3423084

